# Evaluation of percutaneous transcatheter embolization for pulmonary arteriovenous malformations

**DOI:** 10.1186/s12890-021-01448-z

**Published:** 2021-03-05

**Authors:** ZhengZhong Wu, JunQing Lin, WeiZhu Yang, Na Jiang, Ning Huang, Leonardo C. Clavijo

**Affiliations:** 1 Department of Interventional Radiology, Fujian Medical University Union Hospital, 29 Xinquan Road, Fuzhou, 350001 Fujian Province China; 2 Division of Cardiovascular Medicine, Keck School of Medicine, University of Southern California, Los Angeles, CA USA

**Keywords:** Pulmonary arteriovenous malformation, Coil, Plug, Embolization

## Abstract

**Background:**

The purpose of this study was to assess the safety and efficacy of percutaneous transcatheter embolization (TCE) for the treatment of pulmonary arteriovenous malformations (PAVMs).

**Methods:**

Forty-three consecutive patients (n = 17 males; n = 26 females) with 72 untreated PAVMs underwent coil and/or plug embolization between January 2010 and February 2018. The mean patient age was 42 ± 14 years (range 19–71 years). The median size of the feeding artery was 7.9 ± 2.9 mm (range 3.5–14.0 mm). The arterial blood gas level and cardiac function of all patients were analysed. The technical success rate, recanalization rate, and complications were evaluated. Computed tomography angiography (CTA) examinations were scheduled for 12 months after treatment and every 2–4 years thereafter.

**Results:**

Twenty-five PAVMs were treated with coils alone, twenty-one were treated with plugs alone, and twenty-six were treated with both coils and plugs. The technical success rate was 100%. There were no complications during operation. However, one patient (2.3%) had pulmonary thrombosis and embolism post-operation. The patients’ pre-operative and post-operative PaO_2_ and SaO_2_ levels were significantly different (*p* < 0.01). A comparison of the New York Heart Association (NYHA) grade before and after embolization in all patients showed a significant decrease in the post-operative grade (*p* < 0.01). The 72 PAVMs were divided into three groups (coils only group [n = 25], plugs only group [n = 21], and coils/plugs combined group [n = 26]). After 12 months of follow-up, there were seven reperfusion PAVMs in the coil group, seven reperfusion PAVMs in the plug group, and 1 reperfusion PAVM in the combined group. There were significant differences between the two groups and the combined group.

**Conclusion:**

Percutaneous TCE is safe and effective for the treatment of PAVMs. A combination of coils and vascular plugs may be useful for preventing recanalization after the embolization of PAVMs.

## Introduction

Pulmonary arteriovenous malformations (PAVMs) are abnormal structures that represent abnormal communications between an artery and a pulmonary vein that allow blood to bypass the pulmonary capillary bed. Without a capillary network, the pulmonary arterial blood flows directly into the pulmonary venous circulation, resulting in a right-to-left shunt physiologically.

PAVMs are a rare vascular anomaly, with an incidence of only 2–3 cases per 100,000, and the male to female ratio varies from 1:1.5 to 1.8 [1]. Using thoracic computed tomography, Nakayama et al. recently estimated that the prevalence of PAVMs is 38 per 100,000 [2]. More than 80% of PAVMs are congenital, and their precise aetiology is unclear [3]. Common associations are hereditary haemorrhagic telangiectasia (HHT), hepatic cirrhosis, and congenital heart disease. Rare associations include chest trauma, lung parasites, metastatic lung disease, and Fanconi syndrome. Approximately 15–35% of HHT patients in Europe and North America present with PAVMs [4]. Kim et al. suggest that PAVMs are less associated with HHT in Koreans than in Westerners [5]. In a study by Shioya et al., approximately 15% of PAVMs were associated with HHT, suggesting a substantially lower rate in Japan than in Europe and North America. This may be attributed to racial differences or an underestimation in HHT diagnosis [6]. Systemic artery angiography is the gold standard for diagnosing PAVMs. Patients with PAVMs may be asymptomatic but may be at risk for hypoxemia, haemorrhage, and cerebrovascular accidents, such as dyspnoea, haemoptysis, migraine, stroke, and cerebral abscess.

Therefore, it is necessary to treat PAVMs, especially asymptomatic PAVMs. Primary treatments for PAVMs include surgical resection and catheter embolization. The choice is based on the location, number, and size of the PAVMs. However, with the development of interventional technology and materials, catheter embolization is the standard choice [7]. Catheter embolization is a minimally invasive, repeatable treatment, and it does not require general anaesthesia. It also has the advantage of preventing loss of the lung parenchyma.

The aim of the present report was to evaluate the clinical efficacy among 43 PAVM patients who were treated with catheter embolization at our hospital. Furthermore, the effects of different embolization materials were analysed.

## Materials and methods

### Patients

The study group consisted of 43 adult patients treated between January 2010 and February 2018 in the Department of Interventional Radiology, Affiliated Union Hospital of Fujian Medical University. The diagnosis of PAVMs was based on systemic artery angiography, chest computed tomography angiography (CTA) and digital subtraction angiography (DSA). Exclusion criteria included the following: age < 18 years, pregnancy, chronic cardiac or lung disease (such as asthma or chronic obstructive pulmonary disease), suspicion of pulmonary hypertension on echocardiography, lung infection or thromboembolic disease in the past three months, current or past smoker (> 10 packs/year), history of thoracic surgery, and obesity (body mass index > 30 kg/m^2^). All adult patients with PAVMs with diameters ≥ 3 mm identified by our database search who were treated in our centre and for whom imaging scans were performed after the primary embolization procedure were available for study. In accordance with the New York Heart Association (NYHA) classification grading system, the cardiac function of all patients was evaluated. Arterial blood gas was also analysed in all patients. Arterial blood sampling was performed with patients at rest in the sitting position. All operations were performed by a vascular interventional expert with more than 15 years of experience in endovascular therapy and who is proficient in percutaneous catheter embolization.

According to the characteristics and locations of PAVMs, the effective treatment and materials were selected. The diameter of the feeding vessel should not exceed 2 mm using coil only, as to avoid coil migration and incomplete embolization. However, incomplete embolization may exist using plug only, although it could reduce expenses for patients with larger PAVMs. In addition, combined coil and plug may improve the embolization effect and reduce cost.

### Percutaneous embolization procedure

**Fig. 1:**
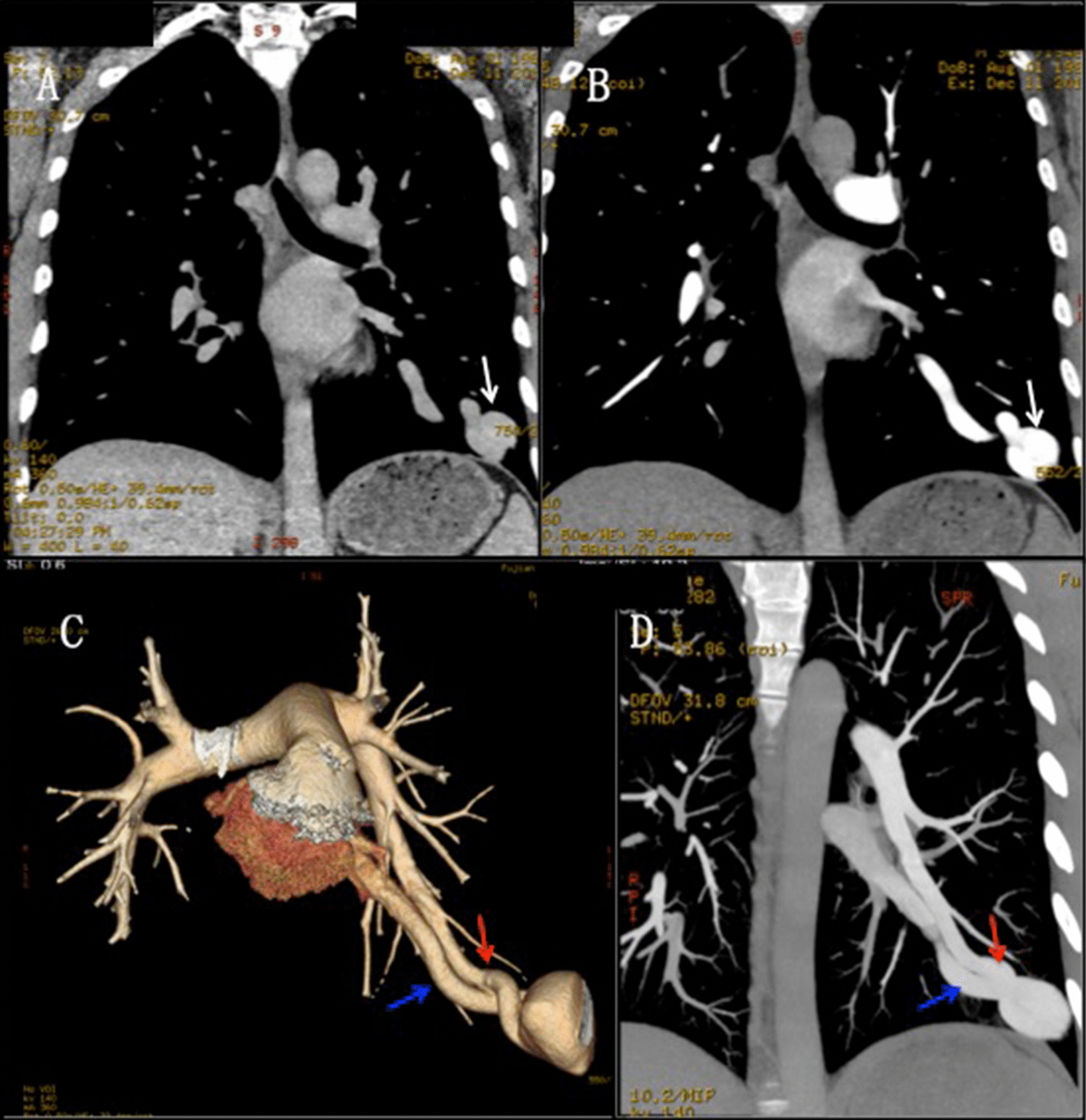
**a** CT scan shows a massive lesion in the left lower lobe (arrow). **b** Enhanced CT shows a massive lesion with suspicion of a vascular malformation (arrow). **c**, **d** CTA and 3-dimensional reconstruction confirm the presence of one PAVM between the lower left pulmonary artery and the pulmonary vein in the left lower lung (red arrow and blue arrow)

Prior to the procedure, all patients underwent CTA to determine the number and location of the PAVMs (Fig. 1). All patients provided informed consent and received adequate pre-operative preparation. Puncture of the right femoral vein using the Seldinger technique was performed under local anaesthesia. After systemic heparin was given through the right femoral vein sheath, pulmonary arteriography was performed to assess the lesion using a 4- or 5-F catheter.

**Fig. 2:**
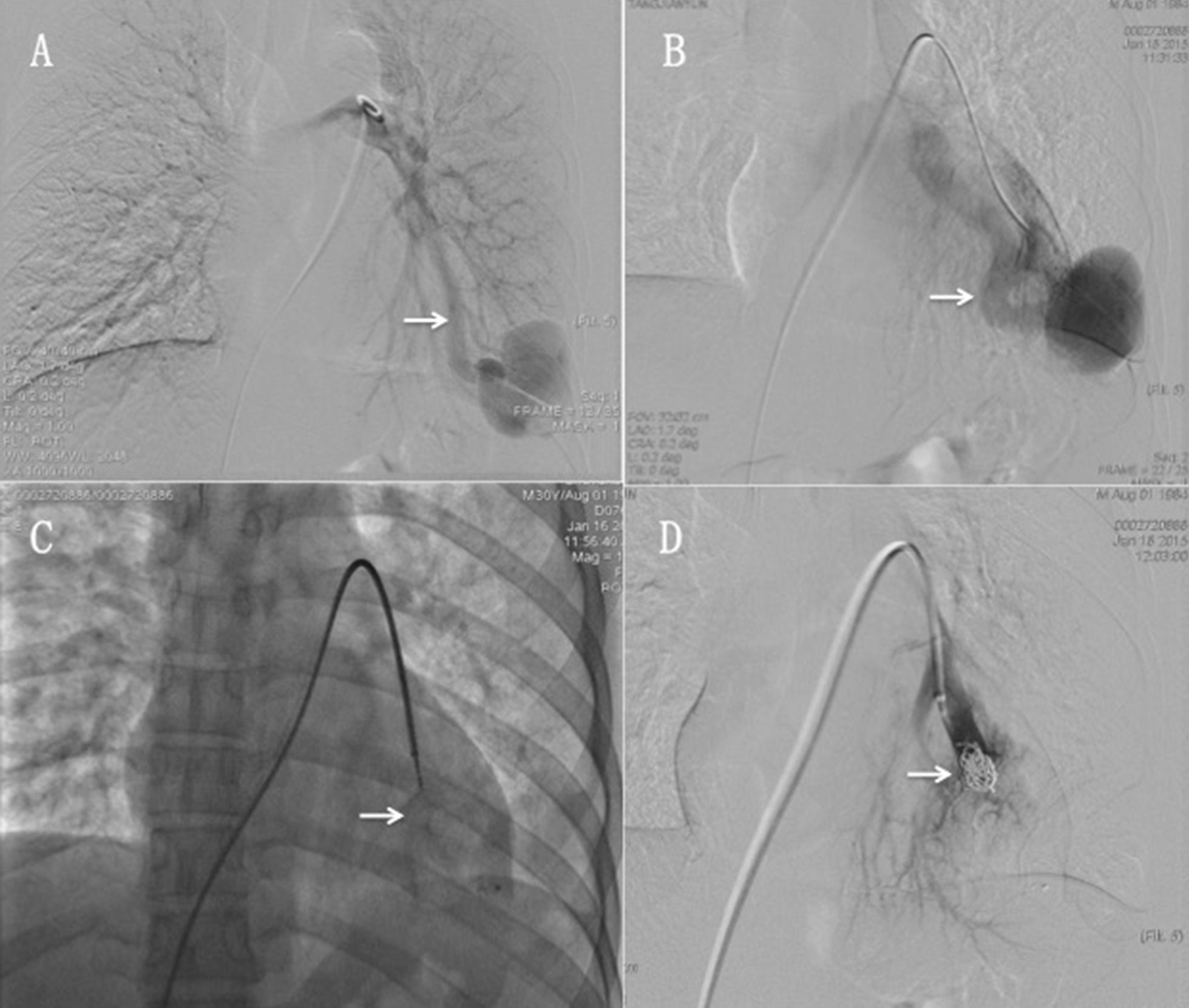
**a** Bilateral pulmonary angiograms show contrast injection directly into the feeding artery (white arrow) with the AVM nidus clearly displayed. **b** Selective angiogram shows an enlarged draining vein (arrow). **c**, **d** Deployment of an Amplatzer vascular plug type I (arrow): a coil was placed (arrow), and contrast was injected into the feeding artery and showed good occlusion, without flow through the PAVMs

First, selective bilateral pulmonary angiograms with an angled pigtail catheter (AP2, Cook Medical, Bloomington, IN, USA) were performed to identify the locations of the PAVMs. Next, we performed selective angiograms using an angled catheter (Berenstein, Cook Medical) to identify the diameters and numbers of feeding arteries targeted for embolization (Fig. 2). Finally, combined with CTA, we formulated embolic methods and selected embolic materials. Available endovascular treatment devices consisted of vascular coils and vascular plugs. No balloons were used.

Transcatheter coil embolization technique [8]: Guided by a 0.035 J type wire (Terumo, Tokyo, Japan), we progressed a Cobra catheter (Terumo) through the right femoral vein sheath to the feeding artery of the PAVMs. If there was any difficulty, a 6-F guiding catheter (Cordis Corporation, Miami Lakes, FL, USA) was used for support. The catheter (or a microcatheter through the catheter) was advanced into the feeding artery of the lesion until its tip was immediately in front of the sac. First, one or more detachable bare microcoils were placed to reduce blood flow. Then, the feeding artery was embolized using pushable fibred microcoils only or a combination of pushable fibred microcoils and 0.035″ coils. After embolization, pulmonary arteriography was performed to confirm complete occlusion of the feeding artery. Typically, a Gianturco coil (Cook Medical) with a diameter of at least 1 mm larger than the feeding vessel was used. The coil anchoring method was performed by radial force using mild oversize. The standard side-branch anchoring technique was not employed since it was felt that this method might occlude normal adjacent vessels.

Transcatheter plug embolization technique [9]: Guided by a 0.035 J type wire (Terumo) through the right femoral vein sheath, we exchanged a 5–6F long sheath (90 cm, Cook Medical) or sent an 8F guiding catheter (Cordis) to the pulmonary artery. Combining the wire with various catheters, we sent the conveying device to the distal feeding artery of the PAVMs. Once the device was deemed to be in a satisfactory position, a vascular plug was deployed, and a single non-subtracted image was obtained. Post-embolization angiography was performed through the guiding catheter at 1-min intervals via manual injection of 5–10 mL of contrast until total occlusion of the artery was visualized.

In case of persistent patency on the post-embolization angiogram for more than 5 min, an additional device was used to achieve complete occlusion (Fig. 2). The diameter of the Amplatzer device was chosen to be at least 20% larger than the diameter of the feeding vessel. Amplatzer vascular plugs (Boston Scientific Corporation, USA) are self-expandable, cylindrical devices made from a nitinol wire mesh secured on both ends with platinum marker bands. A stainless-steel micro screw was welded to one of the platinum marker bands. The device was pushed by a 135 cm pusher wire. In our study, type I Amplatzer plugs with a diameter range of 4–16 mm were used.

Transcatheter combined coil and plug embolization technique [10]: We sent the conveying device to the distal feeding artery of the PAVMs and verified the correct position of the vascular plug by angiography. After confirming the correct position, the plug was deployed into the feeding artery. When recanalization was feared because of the size or complexity of the PAVMs, combined coil embolization was performed in accordance with previous studies. Angiographic control was used to confirm occlusion of the feeding artery and the absence of distal translocation (Fig. 2).

**Fig. 3:**
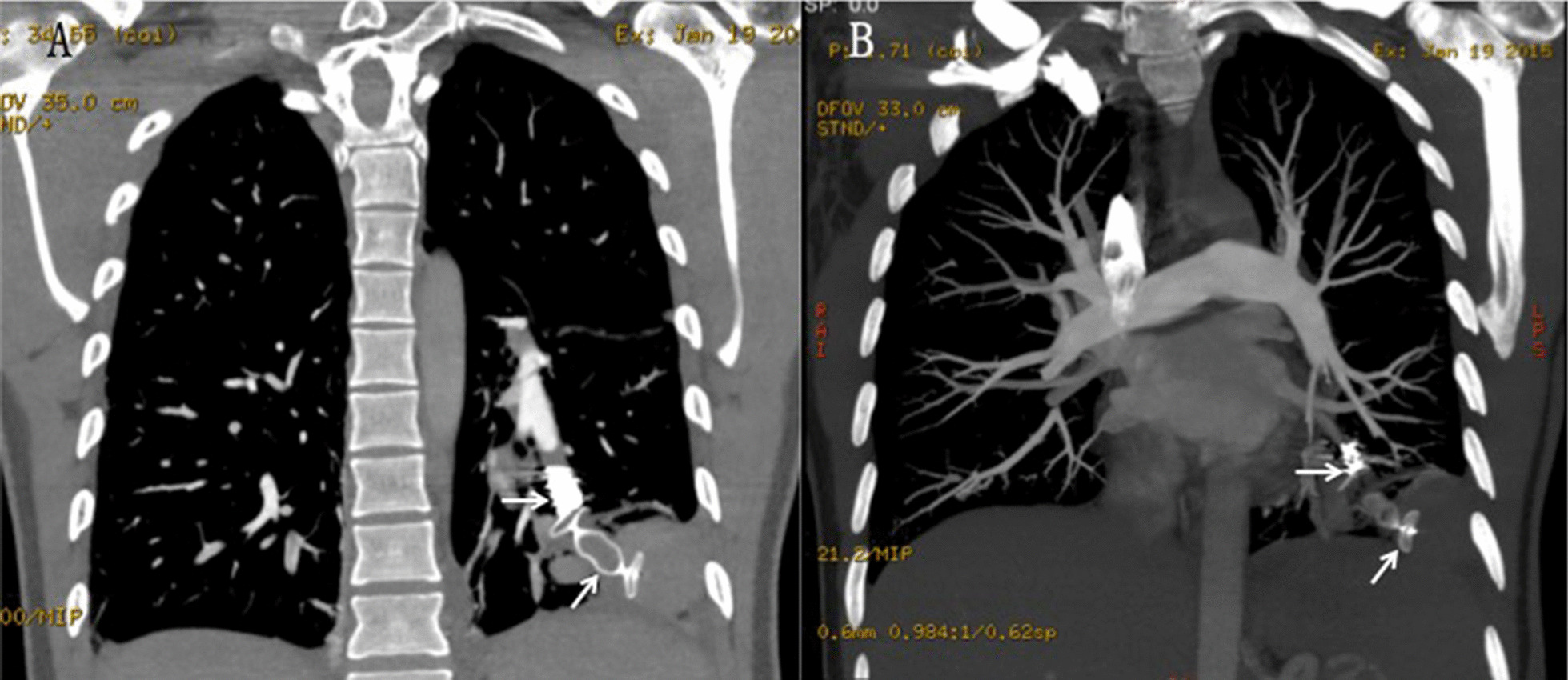
Images from the 6-month follow-up CTA and 3-dimensional reconstruction show that the sac disappeared completely, and the Amplatzer vascular plug and coil (arrow) showed a typical appearance

Prophylactic antibiotics were not used. All patients were discharged to home after 2–3 days of observation in an inpatient ward. The CTA scans were evaluated for perfusion through the embolized segment or beyond and for sac disappearance or shrinkage. Follow-up office visits were scheduled for 1 month and 6 months after treatment. Contrast-enhanced CT was scheduled for the 12-month visit (Fig. 3). Depending on the CT results and determined by the presence or absence of small-untreated PAVMs, long-term follow-up visits were scheduled at intervals of 2–4 years.

Ethics statement: The Ethics Committee of Fujian Medical Union Hospital approved this retrospective study. Written consent was provided by the patients for their information to be stored in the hospital database and used for research.

Statistical analysis: Statistical analysis was performed using the Statistical Package for the Social Sciences (SPSS) software version 18.0 for Windows (SPSS Inc., Chicago, IL, USA). Quantitative data are expressed as the mean ± standard deviation, and qualitative data are expressed as the frequency and percentage. Comparisons of two means were performed using Student’s t-test. One-way analysis of variance (ANOVA) was used to compare three means. ANOVA was used when multiple comparison tests showed significance. Comparisons of frequencies were performed using the Wilcoxon rank-sum test or chi-square test. A *p* value < 0.05 was considered statistically significant.

## Results

The patients’ clinical characteristics are summarized in Table 1. The study group included 43 patients with 101 PAVMs. Based on the abovementioned criteria for treatment, 72 PAVMs were treated with vaso-occlusion. Fifteen patients (34.9%) had single PAVMs, 12 (27.9%) had two PAVMs, and 16 (37.2%) had more than two PAVMs. Thirty-three of the PAVMs (32.7%) were in the right lower lung lobe, 20 (19.8%) were in the right middle lobe, and nine (8.9%) were distributed in the right upper lobe. Thirty-one of the PAVMs (30.7%) were in the left lower lung lobes, and eight (7.9%) were distributed in the left upper lobe. Twenty-five PAVMs (34.7%) were embolized using a coil only, 21 (29.2%) were embolized using a plug only, and 26 (36.1%) were embolized using both a coil and a plug. The immediate technical success rate was 100%, with complete occlusion achieved in all PAVMs.

**Table 1:** Descriptive statistics on patients and PAVMs

	Patient (n = 43)
Age (years): mean ± SD	42 ± 14
Range	19–71
Sex (male: female ratio), n	17/26
Number of patients with HTT, n/N_1_ (%)	23/43
Presentation: n/N_1_ (%)	
Hypoxemia	28/43
Dyspnoea	8/43
Cyanosis	7/43
Cerebral abscess	3/43
Stroke	2/43
Migraine	9/43
Haemorrhage	5/43
PAVMs number (total): n/N_1_ (%)	
Single	15/43
Double	12/43
More than two	16/43
PAVMs location: n/N_2_ (%)	
Right upper lobe	9/101
Right middle lobe	20/101
Right lower lobe	33/101
Left upper lobe	8/101
Left lower lobe	31/101
Treated PAVMs number (N_3_/N_2_)	72/101
Treated PAVMs feeding artery (mm)	7.9 ± 2.9
Range	3.5–14.0 mm
Embolization materials: n/N_3_ (%)	
Coil	25/72
Vascular plug	21/72
Coil and vascular plug	26/72
Complications: n/ N_1_ (%)	
Pleuritic chest pain	5/43
Pulmonary thrombosis and embolism	1/43
Follow-up period (months): mean ± SD	25.02 ± 9.44
Range	12–49

*HTT* hereditary haemorrhagic telangiectasia, *PAVM* pulmonary arteriovenous malformation

**Fig. 4:**
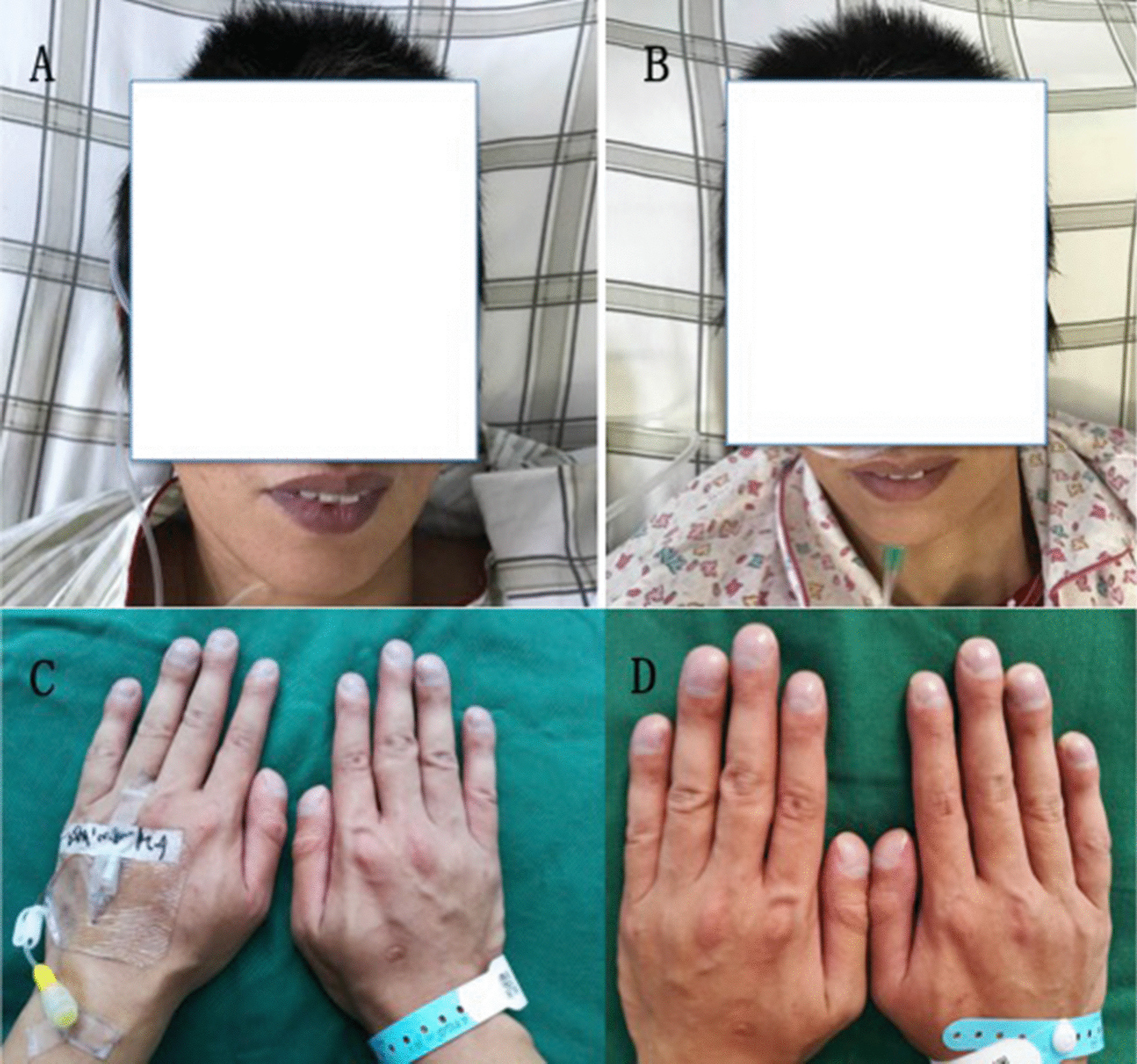
**a**, **b** Pre-operation: the patient had cyanotic lips; post-operation: the lips turned red. **c**, **d** Pre-operation: the patient had cyanotic and clubbed fingers; post-operation: the finger tips turned red

The blood samples was collected form femoral artery at one day pre-operation and three days post-operation without oxygen-inhaling. The pre-operative and post-operative arterial partial pressure of oxygen (PaO_2_) and arterial oxygen saturation (SaO_2_) levels of all patients were significantly different (*p* < 0.01). The arterial partial pressure of carbon dioxide (PaCO_2_) increased post-operation, but there was no significant difference between that pre-operation and post-operation (p > 0.05) (Table 2). Patients’ SaO_2_ levels were > 90% in the absence of oxygen; therefore, one patient with cyanosis experienced notable relief (Fig. 4). A comparison of the NYHA grade before and after embolization in all patients showed a significant decrease in the post-operative NYHA grade (*p* < 0.01) (Table 3). The mean clinical follow-up period was 18 ± 24.3 months (range 12–46 months).

**Table 2:** Arterial blood gas and haemoglobin analyses of 43 patients with PAVMs before and after embolization

	PaO_2_ (mmHg)	PaCO_2_ (mmHg)	SaO_2_ (%)	Hb (g/L)
Preoperation	73.32 ± 10.70	38.21 ± 4.74	89.29 ± 3.97	13.68 ± 1.36
Postoperation	87.71 ± 5.66^a^	38.28 ± 3.85	96.04 ± 1.86^a^	13.13 ± 1.21

Hb, haemoglobin; PaCO_2_, arterial partial pressure of carbon dioxide; PaO_2,_ arterial partial pressure of oxygen; SaO_2_, arterial oxygen saturation

^a^Paired *t*-test showed significant differences between the pre-operation and post-operation values (*p* < 0.01); 1 mmHg = 0.133 kPa

**Table 3:** Comparison of NYHA Grades Before and After Embolization in 43 Patients with PAVMs

	NYHA grade			
	I	II	III	IV
Pre-operation	12	22	9	0
Post-operation	28	11	4	0

Wilcoxon test showed a significant decrease in the post-operative NYHA grade (Z = 3.2998; *p* = 0.0010)

We divided 72 PAVMs into three groups by embolization materials (coil only group [n = 25], plug only group [n = 21], and combined coil and plug group [n = 26]). After 12 months of follow-up, there were seven reperfusion PAVMs in the coil group, seven reperfusion PAVMs in the plug group, and 1 reperfusion PAVM in the combined group. We used the likelihood-ratio chi-square test, which showed significant differences of therapeutic effects between the three groups (X_2_ = 8.8321, p = 0.0121). Using the bootstrap method, we found no significant difference in the recurrence rate between the coil group and the plug group. However, there were significant differences between the two groups and the combined group (Table 4). The mean diameter of the feeding arteries was 7.9 ± 2.9 mm (range 3.5–14.0 mm).

**Table 4:** Efficacy of Different Endovascular Embolization Materials in the Treatment of 72 PAVMs at 12 Months

	Coils	Plugs	Coils and plugs combined
Reperfusion PAVMs	7	7	1
Occluded PAVMs	18	14	25
Total	25	21	26

Likelihood-ratio chi-square test showed significant differences between the three groups (X_2_ = 8.8321; *p* = 0.0121). The bootstrap method revealed no significant difference in the recurrence rate between the coil group and the plug group. However, there were significant differences between those two groups and the combined group

The one-month mortality rate was 0%, and there was no procedure-related mortality. There were no complications during the procedures; however, we encountered 14.0% minor complications in the immediate post-procedure period. Five patients (11.6%) had pleuritic chest pain on the same side of the embolized PAVMs, which was resolved by taking nonsteroidal anti-inflammatory drugs without further management. One patient (2.3%) had pulmonary thrombosis and embolism, which may be attributed to a hypercoagulable state or other factors. This was resolved by using thrombolytic agents and anticoagulants. Furthermore, no injection-related complications were observed.

## Discussion

Currently, minimally invasive transcatheter embolization (TCE) is widely used in the clinic and includes traditional coil embolization technology, detachable balloon embolization technology, occluder embolization technology, etc. TCE with vascular coils and occluders is the standard treatment for PAVMs [10]. Treatment of PAVMs with TCE of the feeding vessels significantly decreases right-to-left shunting, leading to improved oxygenation and relief of clinical symptoms. Due to the location, shape, and characteristics of the feeding and draining vessels of the PAVMs, individualization should be emphasized in the selection of TCE treatment methods and the formulation of treatment plans. Therefore, a pre-operative selective DSA examination of the pulmonary artery is particularly important to determine the location, number of lesions, and specific conditions of the feeding artery and draining vein. For PAVMs with an aneurismal sac lumen diameter of > 2 mm and a feeding artery diameter of > 3 mm, embolization should be performed to avoid serious neurological complications [11].

In this study, the immediate technical success rate was 100%, with complete occlusion achieved in all PAVMs. Minor complications were seen in 13.95% of cases in the immediate post-procedure period. Clinical success was achieved in all patients, with statistically significant increases in PaO_2_ and SaO_2_ levels. A comparison of the NYHA grade before and after embolization in all patients showed a significant decrease in the post-operative NYHA grade. Our study confirms that patients with PAVMs require lifelong clinical and radiological treatment after embolization therapy. We also showed that percutaneous TCE is effective and safe for the treatment of this disease. Percutaneous TCE is recognized as the gold standard for the treatment of PAVMs due to its efficacy and safety in reducing the risk of paradoxical embolism and other complications associated with PAVMs [12–15]. PAVM embolization materials include various coils, detachable balloons, and various occluder devices. The detachable balloon may cause revascularization and ectopic embolization due to its large conveyor, poor controllability and high rate of retraction. Currently, detachable balloons are often used in PAVMs where feeding artery intubation is difficult [16, 17]. Although detachable balloons can achieve the effect and save operating time, we did not use these balloons in any of our patients.

The metal coil is the most widely used embolic material in the treatment of PAVMs. It is suitable for PAVMs where the aneurismal neck is > 3 cm and the blood vessel diameter is tapered at the proximal end of the aneurismal sac. The diameter of the selected coil should be at least 1 mm greater than that of the feeding vessel, and compact embolization should be formed in the feeding vessel as much as possible. The recanalization of PAVMs blocked by coils is a well-known phenomenon, with reported rates of 4–19% [14, 18–22]. Other studies have reported recanalization and reperfusion rates of 25–50% [14, 22–24]. In our study, the rate of recanalization occurring in PAVMs blocked by coils was 28%.

The Amplatzer vascular plug (AVP) is a cylindrical, self-expanding, nickel-titanium memory alloy embolic material that can be repositioned before it is released. It is mainly used for the treatment of congenital heart disease and is currently widely used in the treatment of arteriovenous malformations. The diameter of the device was chosen to be at least 20% larger than the diameter of the feeding vessel. One of the advantages of the AVP is its ability to be recaptured and repositioned. Thus, theoretically, one should be able to position the occluder immediately adjacent to the sac in most instances. Other advantages include its good vascular adhesion and low shift rate and that single release can achieve embolization, which reduces the X-ray exposure time and shortens the operation time. Single AVP embolization is more economical than coil embolization for the same PAVMs. The AVP has a 5–10% rate of recanalization [25]. In our study, the vascular recanalization rate that occurred in PAVMs blocked by AVPs was 33%. A comparison of the use of coils and Amplatzer plugs revealed no significant difference associated with the recanalization rate of the feeding vessels.

However, Amplatzer plugs offer several advantages over traditional coils in the treatment of PAVMs. In the majority of PAVMs, plugs can be placed very distally yet safely in the feeding artery since they can be recaptured and repositioned for better adjustment [26]. This feature is important, since it was documented in a study by Milic et al. [19], who showed that the proximal placement of coils in the feeding artery more than 1 cm from the aneurysm sac was associated with the persistence of PAVMs. Placement of the embolic device in the feeding artery close to the venous sac not only reduces the risk of occlusion of the side branches supplying the adjacent normal lung parenchyma but may also decrease the risk of persistence of the PAVM sac by preventing recanalization of the feeding artery. Distal occlusion is usually very difficult to accomplish when coils are used, especially when the feeding artery is large because of the increased risk of migration of coils into the systemic circulation. Therefore, a combined application may be a good strategy for PAVM treatment.

In our study, 26 PAVMs were embolized with a combination of coils and plugs. We aimed to place the plug in a suitable feeding artery and then aimed to place the coils at the distal feeding artery instead of the venous sac. We believe that tight embolization can be achieved efficiently in the distal feeding artery. Although medical treatment costs are expensive, they are reasonable considering the cost of re-embolization and the burden that is placed on patients. We encountered only one episode of relapse 12 months post-surgery.

The results of this study, which compared the use of coils and Amplatzer plugs in 43 patients with 72 embolized PAVMs, suggest that the combination of coils and plugs is better than plugs alone. We speculate that the polyester fibres on the coils help enhance thrombosis caused by the mechanical effect of the plug. The combination also shows better performance than coils alone. We suggest that this device be used in a manner that minimizes the risks of recanalization and reperfusion.

The incidence of complications following the TCE of PAVMs is low. In addition to severe ectopic embolism, the most common complications are chest pain caused by self-limited pleurisy and pleural effusion, with incidences of 9–31% [18]. There were no complications during the procedures. Five patients experienced pleuritic chest pain on the same side of the embolized PAVMs post-procedure, which was resolved by taking nonsteroidal anti-inflammatory medications without further management. One patient developed pulmonary thrombosis and embolism, which may be attributed to a hypercoagulable state or other factors. This was resolved by using thrombolytic agents and anticoagulants. No ectopic embolism occurred in our study. Long-term complications still include mostly the reperfusion or recanalization of PAVMs. In our study, 15 patients with PAVMs relapsed 12 months post-surgery. There are several mechanisms associated with the persistence, reperfusion or recanalization of PAVMs. One cause of persistent perfusion may be incomplete feeding artery embolization. The mechanism of reperfusion may be recanalization of the embolized blood vessels, thickening of the original tiny feeding artery, or formation collateral circulation of the pulmonary artery, bronchial artery, or other systemic arteries at the distal end of the embolization site. The mechanism of recanalization may be that the feeding artery of the original tiny lesion grows thicker due to haemodynamic changes after embolization of other lesions, and the PAVM lesion increases. Therefore, the following points must be considered before PAVM TCE. First, regardless of the type of embolization material used, a thorough, tight embolus should be formed in the cross-section of the embolized vessel to prevent recanalization. Second, at a timely post-operative angiography, one should carefully observe the embolism completely to avoid missing the tiny feeding artery. Third, one should try to embolize the distal end of the feeding artery to reduce the incidence of collateral circulation. Usually, the residual neck length after embolization should be < 1 cm.

Specific limitations to the study included a relatively small sample size. Because of the retrospective nature of the study, there was an inherent selection bias and inconsistency in the technique of embolization used. Most of the treated PAVMs in this study were simple in angioarchitecture, which does not represent the known incidence in the population. This study represents a single-centre experience, and the results cannot be generalized. Due to severe coil artifact on CT, the embolization of coil might cause false negative for post-operation assessment of recanalization. Other modalities, as angiography from pulmonary artery or time resolved MR angiography, could be employed in the evaluation for recanalization. Comparation of the features of PAVMs between three groups were not performed in current study, it needs more data for analysis. Whether the combined use of coils and plugs can provide excellent long-term occlusion remains to be studied.

## Conclusion

A flexible combined application of coils and plugs for catheter embolization is a safe and effective interventional therapy technology that can improve the symptoms of right-to-left shunts and avoid or reduce neurological complications caused by PAVMs. It also has good mid-term and long-term effects.

## Data Availability

The datasets used and/or analysed during the current study are available from the corresponding author on reasonable request.

## Abbreviations

TCE: Percutaneous transcatheter embolization
PAVMs: Pulmonary arteriovenous malformations
CTA: Computed tomography angiography
NYHA: New York Heart Association
HHT: Hereditary haemorrhagic telangiectasia
DSA: Digital subtraction angiography
ANOVA: One-way analysis of variance
PaO_2_: Pressure of oxygen
SaO_2_: Oxygen saturation
PaCO_2_: Pressure of carbon dioxide
